# Ecological Niche Modeling and Land Cover Risk Areas for Rift Valley Fever Vector, *Culex tritaeniorhynchus* Giles in Jazan, Saudi Arabia

**DOI:** 10.1371/journal.pone.0065786

**Published:** 2013-06-06

**Authors:** Mohamed F. Sallam, Azzam M. Al Ahmed, Mahmoud S. Abdel-Dayem, Mohamed A. R. Abdullah

**Affiliations:** 1 Department of Plant Protection, College of Food Sciences and Agriculture, King Saud University, Riyadh, Saudi Arabia; 2 Jazan University, Jazan, Saudi Arabia; Institut Pasteur, France

## Abstract

**Background:**

The mosquito, *Culex tritaeniorhynchus* Giles is a prevalent and confirmed Rift Valley Fever virus (RVFV) vector. This vector, in association with *Aedimorphus arabiensis* (Patton), was responsible for causing the outbreak of 2000 in Jazan Province, Saudi Arabia.

**Methodology/Principal Findings:**

Larval occurrence records and a total of 19 bioclimatic and three topographic layers imported from Worldclim Database were used to predict the larval suitable breeding habitats for this vector in Jazan Province using ArcGIS ver.10 and MaxEnt modeling program. Also, a supervised land cover classification from SPOT5 imagery was developed to assess the land cover distribution within the suitable predicted habitats. Eleven bioclimatic and slope attributes were found to be the significant predictors for this larval suitable breeding habitat. Precipitation and temperature were strong predictors of mosquito distribution. Among six land cover classes, the linear regression model (LM) indicated wet muddy substrate is significantly associated with high-very high suitable predicted habitats (R^2^ = 73.7%, P<0.05). Also, LM indicated that total dissolved salts (TDS) was a significant contributor (R^2^ = 23.9%, P<0.01) in determining mosquito larval abundance.

**Conclusion/Significance:**

This model is a first step in understanding the spatial distribution of *Cx. tritaeniorhynchus* and consequently the risk of RVFV in Saudi Arabia and to assist in planning effective mosquito surveillance and control programs by public health personnel and researchers.

## Introduction

Thirty-three species of mosquitoes have been reported from the 15 provinces of Kingdom of Saudi Arabia [1]. *Culex tritaeniorhynchus* Giles is a common widespread species in Saudi Arabia recorded from 14 of these provinces, including Jazan. *Culex tritaeniorhynchus* was confirmed as a vector of Rift Valley Fever virus (RVFV) with a biting preference for humans and sheep [2]. Also, this species is a primary vector of Japanese encephalitis (JE) virus in Asia [3]. In 2000, an outbreak of RVF affecting both man and livestock was reported in Jazan Province outside the typical range, Africa [4], [5]. The epidemic was centered in Jazan Province and northern Yemen, and subsequently spread northwards to Asir Province of Saudi Arabia. More than 500 human cases of RVF infection were reported by health-care centers and hospitals in the southern region of Saudi Arabia [6]. Since the 2000 outbreak, only a few additional animal cases have been reported [7].

Globally, *Cx. tritaeniorhynchus* has been implicated in about 50,000 JE cases and 15,000 deaths annually [8]. Although no recent human cases of JE have been reported from Saudi Arabia, a high potential exists. Many laborers from Asian countries where JE is endemic [9] and Africa where RVFV is well established [10] immigrate to KSA in search of job opportunities. The combination of the presence of the JE mosquito vector and possible human viremic sources has sustained public health concerns in Saudi Arabia. The significant mortality and morbidity caused by infectious mosquito borne diseases in the developing world [11] promotes development of regional/international vector control efforts to reduce disease implications. These applied control efforts are dependent on the spatial distribution of the mosquito vector species [11]–[13]. Geographic information systems (GIS) and remote sensing (RS) allow the ability to detect mosquito larval breeding habitats and forecast their distribution [14], [15]. The ability to use such techniques in predicting high-risk areas for mosquito vector populations is based on the relationship between ecological, topographic and/or geographic variables [16]. Such technologies have been successfully used to predict the mosquito vector densities [17], [18], and classify the risk of vector and vector borne disease transmission [11], [19]–[22].

The ecological niche modeling program, Maximum Entropy (MaxEnt), was used in the analysis and development of a predictive spatial model for the distribution of *Cx. tritaeniorhynchus*. Such modeling techniques have been previously used to predict the spatial distribution of mosquito vectors [20]. Understanding the spatial distribution of *Cx. tritaeniorhynchus* may allow implementation of preventive measures against this vector. Modeling the spatial distribution of mosquito vectors is dependent on environmental factors including temperature, precipitation, and elevation [23], [24]. GIS/RS technology and environmental niche models such as MaxEnt may help in better understanding how these factors are driving mosquito vectors.

In southern provinces of Saudi Arabia, previous investigations have discussed the occurrence records and morphological classification of mosquito species [1], [25] or addressed the incidence of mosquito borne diseases such as RVF virus in Jazan [2], [26], [27]. The previous studies conducted in Jazan Province failed to describe the spatial preference of larval *Cx. tritaeniorhynchus* breeding sites. This is the first study in Saudi Arabia to define the land cover and ecological variables contributing to mosquito larval habitats.

In the current work, 22 bioclimatic and topographic layers with a resolution of ∼1 km and ecological niche model software (MaxEnt) was used to characterize the larval suitable habitat requirements of *Cx. tritaeniorhynchus* in Jazan Province. Also, a supervised classified image of SPOT5 satellite, with a fine resolution of 2.5 m, was used to assess the land cover distribution in 2 km buffer zone around the collection sites within the suitable predicted habitat derived from MaxEnt. This allowed an opportunity to develop a predictive spatial distribution for suitable mosquito habitat and the land cover contribution within this niche. This may benefit in risk assessment and vector control programs.

## Methods

### Study Area

**Figure 1:**
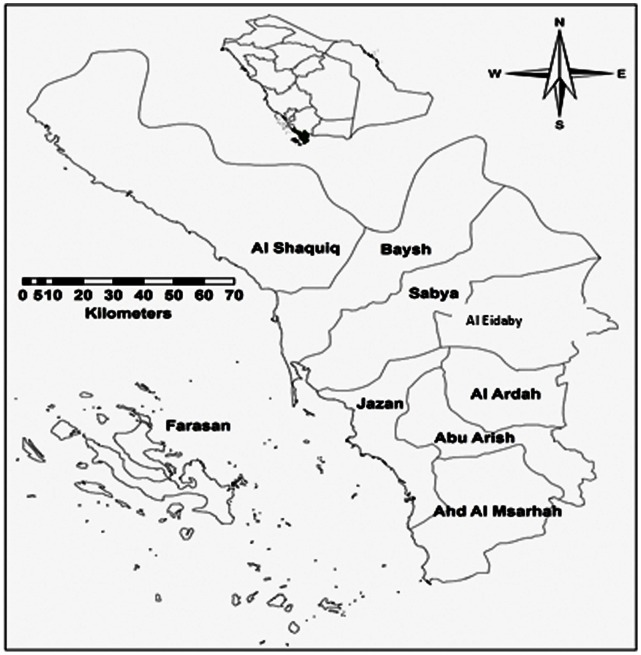
Map of Jazan Province and its political districts.

**Figure 2:**
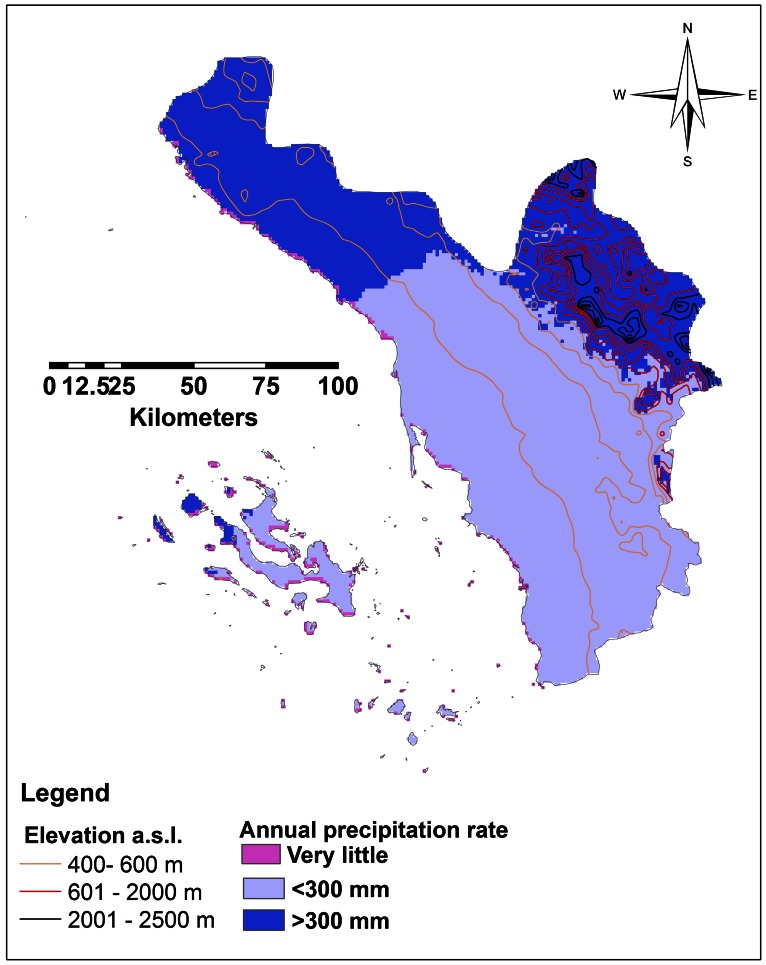
Map of Jazan Province representing different elevation levels and annual precipitation rate.

Kingdom of Saudi Arabia (KSA) occupies approximately 2,250,000 km^2^ of the Arabian Peninsula with a variable topography including areas of arid, semiarid, and forested land scape. Saudi Arabia has a population of about 27 million distributed in 13 provinces, including about 11 million recent immigrants, including almost two million illegal residents [28], [29].The current study was conducted in Jazan Province, which is located in south-western Saudi Arabia (Fig. 1) and includes ∼13,432 km^2^ [30] inhabited by 1,365,110 people averaging 117people/km^2^ [28], [30]. The Red Sea forms the western boundary; the Republic of Yemen forms its eastern and southern boundaries, and Aseer region is the northern border [31]. Topology and climate of Jazan can be categorized into three distinct sectors: the eastern Sarawat Mountains range 2,000–2,500 m a.s.l. with an annual precipitation rate >300 mm; hilly middle areas north to south with elevation range 400–600 m a.s.l. and <300 mm rain/year; and the coastal western plains with elevation <400 m a.s.l. and little, if any, annual precipitation (Fig. 2). Overall the Province has typically two rainy seasons, May-July and September-November.

**Figure 3:**
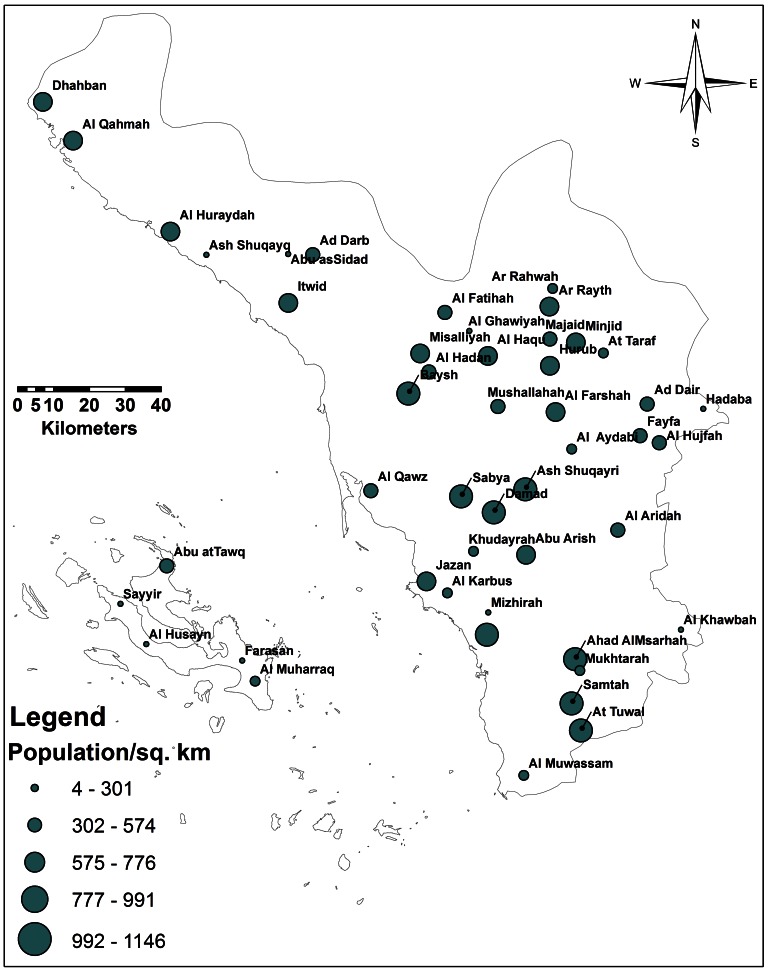
Map of Jazan Province representing distribution of human population.

Jazan is divided into nine administrative districts (Fig. 1): Al Shaquiq (69,134 people/3632 km^2^), Baysh (774,421 people**/**827 km^2^) Sabya (22,8375 epeopl**/**1,983 km^2^), Al Eidaby (60,799 people/1,290 km^2^), Abu Arish (197,112people**/**927 km^2^), Al Ardah (76,705people**/**852 km^2^), Jazan (157,536 people/887 km^2^), Ahd Al Msarhah (110,710 people/1,348 km^2^), and Farasan (17,999 people/686 km^2^) (Fig. 3).

### Mosquito Collection and Sampling Frame

This study was part of a regular surveillance program. During the survey, all accessible permanent water bodies were sampled. The term of “permanent water body” in this study refers to any discrete body of water that is likely to remain inundated for at least 7 days. To improve the environmental sampling, larval mosquito collections were taken from different landscapes features including Mountains, Hills, and Coastal areas across Jazan Province. Mosquito larvae were collected for 60 days during February and May, 2012. Samples were collected using standard 350 ml plastic dippers. Larval collection sites were recorded using global positioning system (GPS) (Garmin, eTrix®). In the laboratory, fourth-instar larvae were mounted on glass slides using Puri’s media. Species were identified using Al Ahmad et al. [1]. Positive larval records for collection sites of *Cx. tritaeniorhynchus* were saved as CSV format for use in MaxEnt. Physiochemical parameters such as pH and total dissolved salts (TDS) for all collection sites were recorded using pH/TDS testers (HANNA Instruments, USA) for further statistical analysis.

### Geographic Information System (GIS)/Remote Sensing (RS)

To characterize suitable larval habitat requirements of *Cx. tritaeniorhynchus* and model its spatial pattern, nineteen bioclimatic variables (11 layers of temperature and 8 precipitation indices) and elevation layer were obtained from the WorldClim database ver.1.4 (www.worldclim.org) [32]. These layers are available at a 30 arc-seconds (∼1km) resolution. The layers were then clipped to match dimensions of the Jazan Province and saved as ASCII grids using Model Builder in ArcGIS software v.10. The aspect ratio and slope were generated from a 30 arc-seconds digital elevation model (DEM) using the surface spatial analyst tool in Arc tool box of ArcGIS ver. 10 and saved as ASCII grids. A total of 22 clipped ASCII layers and the mosquito larval occurrence records were then used to predict the habitat suitability of *Cx. tritaeniorhynchus* in Jazan Province using MaxEnt software v.3.3.

To assess the distribution of land cover classes in 2 km buffer zone around the collection sites within the suitable predicted habitat derived from MaxEnt, eleven orthorectified SPOT5 scenes with 2.5 m resolution were acquired for the years 2010 and 2012. Image scenes were mosaicked and cropped to the dimensions of Jazan Province using ERDAS Imagine software Ver. 9.1. SPOT images were georeferenced to a UTM projection with a WGS-84 datum by using ground control points (GCP) collected during the pilot field survey with a hand-held GPS unit. Almost cloud-free images were available during the dry season (January-March). This helped in reducing the influence of phenological differences and classification errors. Radiometric correction was carried out on the satellite images to reduce the haze and equalize histograms using ERDAS Imagine software. A maximum likelihood supervised classification with 6 statistically distinguishable classes representing different land cover classes was performed. The land cover classes were urban, vegetation (native trees, shrubs, and agriculture), wet muddy substrate, water covered with vegetation, sandy soil, and barren land. For land cover classes, 240 and 47 pixels were randomly selected to be used as regions of interest (ROIs) for training and testing the classification, respectively. The classified satellite image was saved in ERDAS Imagine as an IMG file.

The land cover distribution in 2 km buffer zone around collection sites was categorized into three risk levels: very low-low, medium and high-very high. The land cover distribution was categorized on the basis of predicted habitat suitability values from MaxEnt using ArcGIS ver. 10 and saved as shape file. The shape file format gives the chance to divide the collection sites within 2 km buffer zones according to the MaxEnt-derived risk classes, and subsequently to examine the land cover distribution at these sites based on predicted risk classes.

### Ecological Niche Model of *Culex tritaeniorhynchus*

Maximum Entropy (MaxEnt) software v. 3.3 [33]–[35] was used to model the ecological niche of *Cx. tritaeniorhynchus* over the study area. The software uses only the occurrence records of the mosquito species in conjunction with other bioclimatic attributes to predict the habitat sites suitability for larval breeding. The software was configured to the “Auto Features” mode as suggested by Phillips and Dudik [35], the logistic output format, and ASCII output file type. The model was run without background data, since the bioclimatic layers used in the model were clipped to Jazan Province and have the same dimension and resolution. MaxEnt reduces the duplicate records within ∼1 km of the same cell size [36]. Records of larval mosquito vector were randomly partitioned for model evaluation into two subsamples: 75% of the records used for training and building up the model, and the remaining records (25%) were used for testing the model’s accuracy. In this model, two indicators have been used to examine the performance accuracy. Extrinsic omission was evaluated at fixed threshold (10 percentile training presence) and the area under the curve (AUC) of the receiver operating characteristics (ROC). Although MaxEnt does not require species absence data for model training, absence data was generated to validate the predictive models. The absence dataset was based on our field collection during the current study. The contribution of the environmental variables to the model was estimated using the Jackknife analysis in MaxEnt software. A total of 22 bioclimatic and topographic layers (Table 1) were used to predict the habitat suitability, and spatial distribution of *Cx. tritaeniorhynchus.* In addition, the contribution of these variables in our model was evaluated. To represent the spatial range of the mosquito vector, the predicted habitat probability was categorized into five classes; very low (0–0.1), low (>0.1–0.2), medium (>0.2–0.4), high (>0.4–0.6), and very high (>0.6) using natural breaks in the symbology tools in ArcGIS software v.10 (Fig. 2).

**Table 1:** Twenty two variables used in the MaxEnt niche model to predict the distribution of *Culex tritaeniorhynchus.*

Variable	Description	% contribution to the model
**bio18**	Precipitation of Warmest Quarter	33.4
**bio13**	Precipitation of Wettest Month	21
**bio8**	Mean Temperature of Wettest Quarter	18.3
**bio15**	Precipitation Seasonality (Coefficient of Variation)	9.1
**bio19**	Precipitation of Coldest Quarter	8.3
**bio14**	Precipitation of Driest Month	4.8
**Slope**	Slope	2.2
**bio16**	Precipitation of Wettest Quarter	1.4
**bio12**	Annual Precipitation	0.7
**bio9**	Mean Temperature of Driest Quarter	0.3
**bio2**	Mean Diurnal Range (Mean of monthly (max temp - min temp))	0.3
**Bio1**	Annual Mean Temperature	0
**Bio3**	Isothermality (BIO2/BIO7) (* 100)	0
**Bio4**	Temperature Seasonality (standard deviation *100)	0
**Bio5**	Max Temperature of Warmest Month	0
**Bio6**	Min Temperature of Coldest Month	0
**Bio7**	Temperature Annual Range (BIO5-BIO6)	0
**Bio10**	Mean Temperature of Warmest Quarter	0
**Bio11**	Mean Temperature of Coldest Quarter	0
**Bio17**	Precipitation of Driest Quarter	0
**Aspect**	Aspect ratio	0
**Alt**	Altitude in degrees	0

### Field Validation of the Model

A field survey was carried out for 30 days during June and November, 2012 to evaluate the suitable probabilities of several sites and to compare these with probability values from the model. For this field validation we visited 72 sites that presented different predicted habitat suitability values. We used the predictive map generated from the model to identify sites for validation. The selected collection sites were visited using GPS navigator.

### Statistical Analysis

To model the abundance of *Cx. tritaeniorhynchus* larvae based on water physiochemical variables (pH/TDS), and to determine the predicting land cover type within suitable predicted habitat, linear regression analysis (LM) was carried out using Enter method. The abundance of mosquito larvae collected from field validation points was also modeled in relation to different predicted risk probability using LM, and the difference in mean larval density in regard to predicted risk probability was compared using analysis of variance test (ANOVA).The statistical analysis was performed using SPSS package ver. 17 (SPSS Inc., Chicago, Illinois, US).

### Ethical Approval

The current work was conducted under the auspices of King Saud University, Riyadh, KSA, and permission was obtained from the Municipal Government in Jazan, Malaria and Public Health Department, and Department of Agriculture in Jazan.

## Results

**Figure 4:**
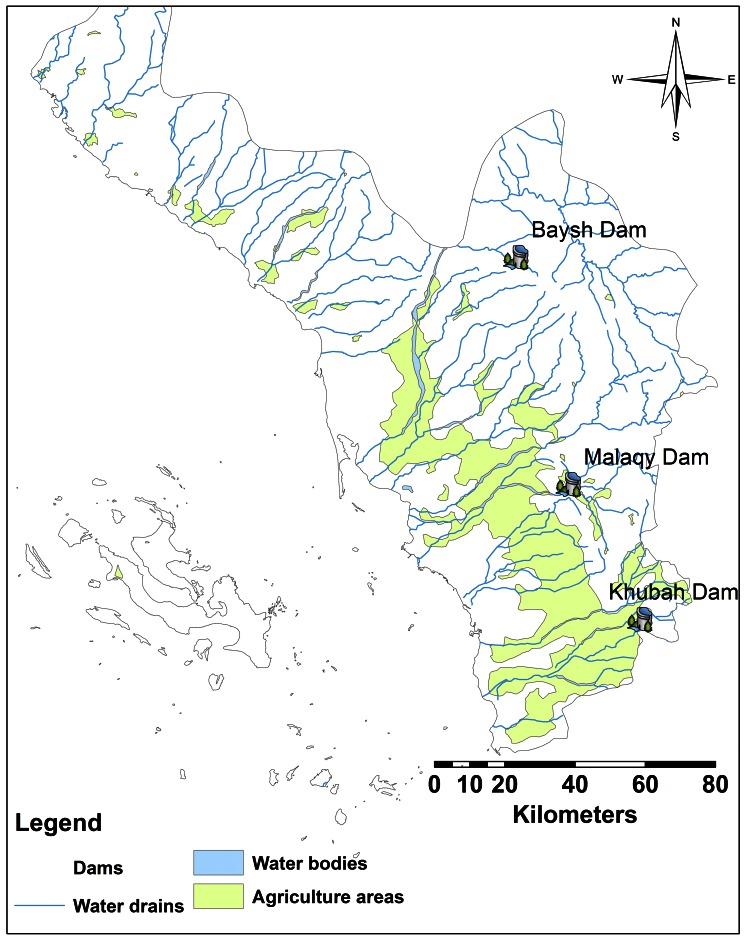
Map of Jazan Province representing water drains/bodies, dams’ locations and agricultural area.

A total of 3,090 mosquito larvae were collected in 60 days in Jazan Province from 98 sites of different elevations during the dry season from February-May, 2012. Of the 98 sites sampled, 78.6% (77 water bodies) were positive for mosquito larvae. *Culex tritaeniorhynchus* and *Cx. sinaiticus* Kirkpatrick comprised 31.2% and 36.1%, respectively of the total number of larvae collected. *Culex tritaeniorhynchus* larvae were found in 51% (50 water bodies) of the sites. The aquatic habitats sampled were categorized into six types including burrow pits, rain pools, stream bed pools, irrigation ditches, water tanks, and open lakes formed by dams (Fig. 4).

The linear regression model used to predict *Cx. tritaeniorhynchus* abundance in regards to the pH/TDS variables was significant (*P<*0.01). Nevertheless, R^2^ value indicates very low correlation between mosquito vector larvae and these physiochemical predictors (R^2^ = 23.9%). The TDS was shown to be a significant contributor in detecting mosquito larvae (*P<*0.01), whereas pH variable did not improve the model (*P>*0.05).

### Ecological Niche Model of *Culex tritaeniorhynchus*

**Figure 5:**
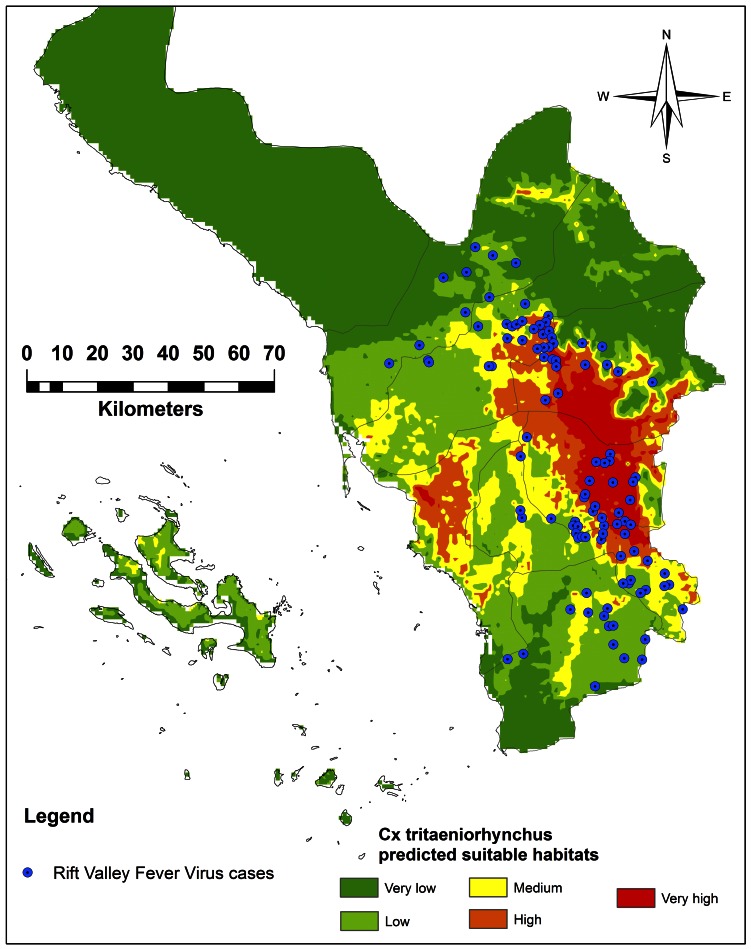
Ecological niche model of *Culextritaeniorhynchus* and RVF human cases recorded during 2000 [43] in Jazan Province, Saudi Arabia.

The model was performed using 75% (n = 35) of points for training and 25% (n = 11) for testing. The predictive performance was found high with an AUC value of 0.936 and 0.914 for train and test occurrence records, respectively, with a standard deviation of 0.025. The fractional predicted area, at 10 percentile training presence was 0.216 and the test points omission rate was 0.091. These points were classified as significantly better than random (*P*<0.0001). MaxEnt predicts 670.43 km^2^ of very high suitable habitat (predicted risk probability >0.60), which is 4.99% of the total area of Jazan Province (Fig. 5).

**Figure 6:**
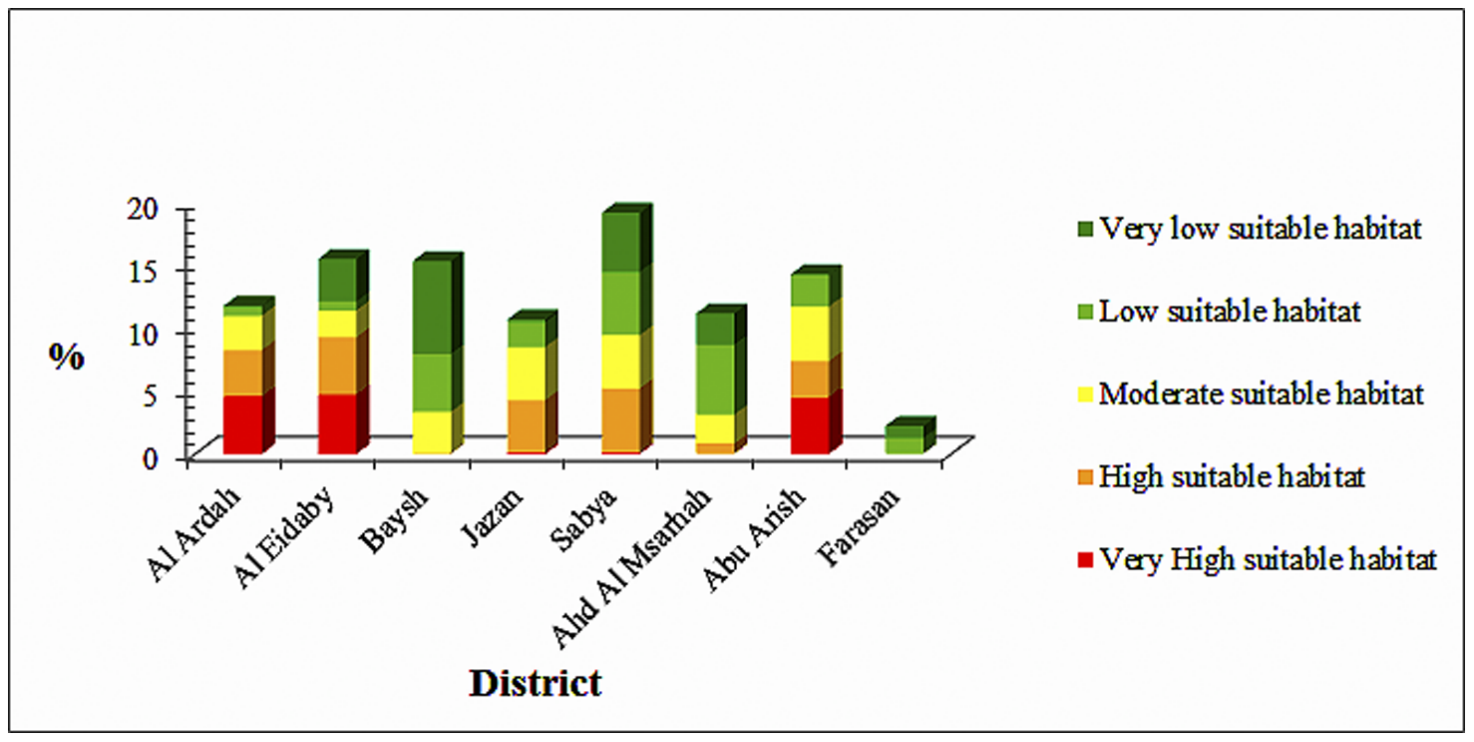
Relative distribution of predicted suitable habitats in different political districts of Jazan Province, Saudi Arabia.

Generally, the MaxEnt created model for *Cx. tritaeniorhynchus* supported three topographic sectors representing different elevations ranges from 30–2,000 m. The very high predicted suitable habitat was found to be spotty in two main sectors with different elevations. The prediction map indicated consistent major distribution of very high suitable predicted habitats in hilly middle and eastern Sarawat Mountains near the cities of Abu Arish, Al Eidaby, Sabya, Ahd Al Masarhah, and Al Ardah. The elevation range of these cities varied from ∼600–2,000 m a.s.l. The predicted habitat suitability across this region represented 652.42 km^2^ (97.31%) of the total very high suitable predicted habitats. These very high suitable predicted habitats were consistent with the incidence of RVFV human cases that have been previously recorded during the outbreak of 2000 (Fig. 5). However, no recent human cases have been recorded to be compared with our model. Also, another 18.01 km^2^ (2.69%) was indicated as very high suitable predicted habitat along the western coast of the Jazan Province at an elevation range 0–30 m (Fig. 6).

### Contribution of the Variables to the Model

Among the 22 bioclimatic and topographic variable layers used for spatial predictions of *Cx. tritaeniorhynchus* using MaxEnt software, 11 variables were found to contribute in species spatial prediction (Table 1). The Jackknife test showed the precipitation variables significantly improved predictive power (78.7%) with the highest training gain compared to other environmental variables. The precipitation of the warmest quarter variable (bio18) presented the utmost training gain in the model. Furthermore, the temperature related variables shared a significant reduced training gain (18.9%) with precipitation in the model. The surface slope had the smallest effect (2.2%), compared to precipitation and temperature.

The land cover distribution in 2 km buffer zones around collection sites within the suitable predicted habitats showed that both sandy soil and barren land constitute the greatest ratios of the land cover classes, 38.83% and 38.31%, respectively. Wet muddy substrate, vegetation, and water bodies covered with vegetation (streams, rain drains, and water pools) represented 13.53%, 4.95%, and 3.88%, respectively. Whereas, the urban land cover contributed the lowest ratio (0.50%) in these buffer zones.

**Figure 7:**
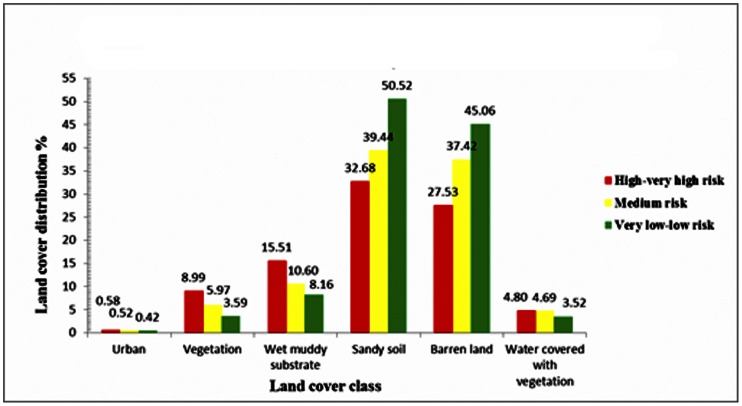
Relative distribution of land cover within predicted suitable habitats of *Culextritaeniorhynchus* in Jazan Province, Saudi Arabia.

The linear regression model represented insignificant correlation (*P*>0.05) between the pixel numbers of land cover types and risk probability classes (high-very high, medium, and very low-low). Nevertheless, the statistical model showed significant positive correlation between wet muddy substrate and risk level (*P*<0.05). The R^2^ value (73.7%) indicated that suitable predicted habitats are significantly depend upon the wet muddy land cover distribution. Therefore, the increase in ratio of wet muddy substrate within high-very high risk regions increases the risk for *Cx. tritaeniorhynchus* larval breeding (Fig. 7).

### Field Validation of the Model

**Figure 8:**
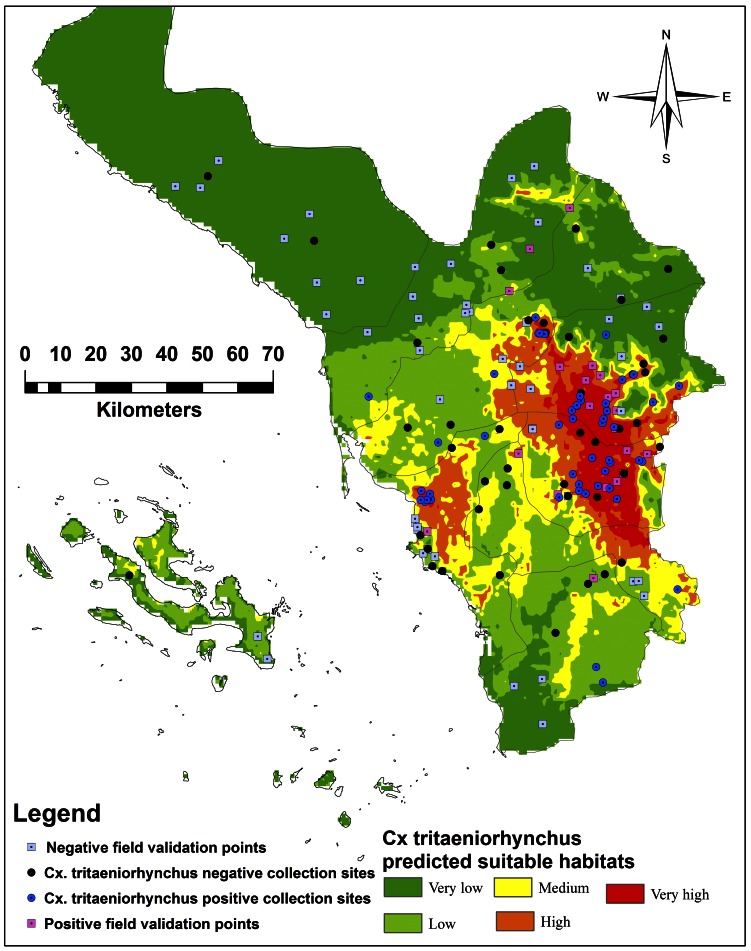
Field collection and validation sites compared to ecological niches of *Culextritaeniorhynchus* in Jazan Province, Saudi Arabia.

Twenty-two sites (30.6%) were positive for *Cx. tritaeniorhynchus* larvae, whereas fifty (69.4%) were negative (Table 2). Of these positive collection sites, eleven (100%) in very high, six (45.45%) in high, three (30%) in medium, two (13.13%) in low, and one (4%) in very low risk predicted areas were recorded. The negative collection sites were found to be in water bodies with high TDS values in eastern moderate-high mountain regions and/or in plateau coastal low elevation areas in the western regions representing Jazan City, Farasan Island, western Beish and western Ahd Al Masarhah. The suitable predicted habitats for mosquito vector abundance were concentrated in high and moderate elevations (600–2,000 m) at the Sarawat eastern mountain range and hill middle areas (Table 2, Fig. 8). A few suitable predicted habitats were found in western regions with TDS values lower than 1 ppm (*P*<0.05).

**Table 2:** Field validation for the ecological niche model of *Culex tritaeniorhynchus.*

Predictedprobability	Coordinates		Field evaluation result	Mosquito larvae abundance*
	Longitude	Latitude		
**>0.6**	43.04	17.271	+ve	98
	43.08827	17.13516	+ve	191
	42.994	17.249	+ve	501
	43.063	17.057	+ve	89
	42.879	17.432	+ve	53
	42.918	17.348	+ve	63
	42.984	17.314	+ve	85
	43.06	17.281	+ve	83
	43.06	17.192	+ve	99
	43.08	17.077	+ve	71
	43.037	17.042	+ve	79
**>0.4–0.6**	43.001	17.351	+ve	88
	43.556	17.03	+ve	67
	42.913	17.024	+ve	76
	42.813	17.128	+ve	61
	42.848	17.191	+ve	221
	42.849	17.19	−ve	0
	42.844	17.292	−ve	0
	42.795	17.301	−ve	0
	42.817	17.349	−ve	0
	42.773	17.368	−ve	0
	42.833	17.46	−ve	0
**>0.2–0.4**	42.79	17.54	+ve	11
	43.00815	17.309	+ve	18
	43.039	17.685	+ve	21
	42.601	16.867	−ve	0
	42.93929	17.01996	−ve	0
	42.68814	17.48722	−ve	0
	42.67801	17.48428	−ve	0
	42.56302	17.3894	−ve	0
	42.61396	17.26542	−ve	0
	42.57154	16.87514	−ve	0
**>0.1–0.2**	43.062	17.237	+ve	10
	43.003	16.812	+ve	8
	43.14092	17.50007	−ve	0
	42.54989	16.93167	−ve	0
	42.5469	17.93422	−ve	0
	42.54163	16.94825	−ve	0
	42.53845	16.95578	−ve	0
	42.5817	16.8846	−ve	0
	42.989	17.598	−ve	0
	42.176	16.607	−ve	0
	42.152	16.664	−ve	0
	43.10414	16.80369	−ve	0
	43.11797	16.80466	−ve	0
	43.13197	16.7605	−ve	0
	43.07466	17.37439	−ve	7
**0–0.1**	42.842	17.647	+ve	0
	42.64184	17.60909	−ve	0
	42.551	17.601	−ve	0
	43.16955	17.4494	−ve	0
	43.07164	17.52286	−ve	0
	42.41206	17.56723	−ve	0
	42.283	17.73533	−ve	0
	41.943	17.806	−ve	0
	42.053	17.871	−ve	0
	42.219	17.673	−ve	0
	42.302	17.562	−ve	0
	42.326	17.481	−ve	0
	42.43	17.436	−ve	0
	42.545	17.526	−ve	0
	42.681	17.505	−ve	0
	42.796	17.827	−ve	0
	42.853	17.857	−ve	0
	42.863	17.715	−ve	0
	42.873	16.556	−ve	0
	42.802	16.538	−ve	0
	42.875	16.442	−ve	0
	43.043	17.469	−ve	0
	42.006	17.802	−ve	0
	42.56	17.471	−ve	0
	43.07372	17.23565	−ve	0

* LM (R^2^ = 31.1, *P*<0.01). ANOVA (F = 11.78, df = 4, *P*<0.01).

The LM suggested that abundance of mosquito larvae is dependent on the suitable predicted habitats (R^2^ = 31.1, *P*<0.01). Also, ANOVA showed significant differences in the abundance of mosquito larvae collected from different predicted risk probabilities (F = 11.78, df = 4, *P*<0.01).

## Discussion

In the last two decades many investigations have demonstrated that GIS/RS are important tools in producing spatial predictive maps to give better understanding of the ecological factors affecting infectious diseases transmission [22]and to study the spatial and temporal patterns of vector borne diseases [20], [21].

Using niche model tools may facilitate the characterization of *Cx. tritaeniorhynchus* larval breeding sites, and ultimately predicting the spatial risk of their suitable habitats. This may help in producing risk assessment maps and in implementing targeted control measures. GIS/RS tools were successfully used in mapping mosquito vectors breeding sites along river valleys for other regions [37], [38].

The current investigation is the first to use GIS/RS to model the spatial distribution of *Cx. tritaeniorhynchus* microhabitats in KSA. Little information was previously available regarding the study area, site of previous RVFV outbreaks, concerning the spatial characterization of larval mosquito microhabitats and mapping areas of mosquito borne diseases risk. We built a niche model for *Cx. tritaeniorhynchus* in Jazan Province, KSA using most recent field data set on species occurrence. We also evaluated the model using independent field data and field validation. Then a model was developed in the framework of MaxEnt [35]. Our model examined the spatial heterogeneity in *Cx. tritaeniorhynchus* larval breeding sites and resulting vector risk distribution in RVFV endemic areas. This study elucidated the dependency of *Cx. tritaeniorhynchus* on lagged environmental variables (land cover, temperature, precipitation) in Jazan Province. Such dependency has been reported by other investigations [23], [24], [39], [40]. Preferred larval habitats are dependent in part on mosquito biology; larvae of some species are also better adapted to specific water chemistry or specific habitats [41], [42].

In our study, natural water bodies were mapped using updated satellite SPOT5 images. These sites were located in mountains, hilly areas, and valleys associated with human settlements. The supervised classification of the satellite images gave recent depiction of the current land cover classes in the study site, allowing the assessment of the contribution of land cover classes in high suitable predicted habitats of *Cx. tritaeniorhynchus* [22].

The results of the modeling effort are consistent with the available literature on *Cx. tritaeniorhynchus* distribution in Jazan [2], [26], [27], [43]. Also, the very high and high suitable predicted habitats in the Sarawat Mountains and hilly middle areas were consistent with the incidence of RVFV human cases that had been reported during the outbreak of 2000 in Al Ardah, Sabya, Al Eidaby, Abu Arish, and Ahd Al Msarhah Districts [43]. However, no recent RVFV cases were available to be included in our model. Additionally, since the 2000 outbreak, there have been changes in land cover of the area sampled. These changes were not accounted for in our study. Further investigations will be conducted to study how land cover changes have influenced mosquito populations.

It is well known that global climates parameters such as temperature and precipitation are the proxy attributes regulating mosquito density [39], [40]. The influence of each environmental data layer on the model was evaluated via a Jackknifing procedure. The environmental attributes related to temperature and precipitations have been confirmed to significantly influence *Cx. tritaeniorhynchus* occurrence/distribution [23]. Precipitation of warmest quarter provided the largest contribution in terms of predicting *Cx. tritaeniorhynchus* occurrence. The highest probability was in areas with highest precipitation as occurring in the Eastern Sarawat mountain ranges (2,000–2,500 m a.s.l.) and hill middle areas (400–600 m a.s.l.) with annual precipitation rate >300 mm and <300 mm, respectively. This is not surprising as higher precipitation increased the filling of water bodies for larval breeding [44]. Although *Cx. tritaeniorhynchus* is occasionally collected above 1,000 m [45],[46], additional sampling will be needed to determine if this is true in Jazan Province. The main variable that may limit the distribution of *Cx. tritaeniorhynchus* in these areas is the drainage of slopes. Human modified land cover such as water retention construction (terracing, burrow pits, water tanks, irrigation ditches) in higher aspect slopes may provide larval mosquito breeding sites.

We found a significant relationship between larval abundance and total dissolved salts (TDS). This finding is consistent with previous studies, where low TDS values are preferred by *Cx. tritaeniorhynchus* [41], [42].

The land cover distribution in 2 km buffer zones around collection sites within suitable predicted habitats was not surprising, as *Cx. tritaeniorhynchus* larvae significantly associated with wet muddy land cover. This finding is consistent with other investigations that concluded sandy and barren land cover results in a low risk level, whereas wet muddy soil correlates with high risk levels [47], [48]. The supervised classification of land cover classes in Jazan Province revealed fragmented landscape consisting of wet muddy substrate, vegetation and urbanized areas predicted the occurrences of *Cx. tritaeniorhynchus* larval habitats.

The field validation collection points for our model demonstrated that the percentages of the positive sites corresponded with the predicted suitability values of the model. This can be attributed to the suitable niches that include wet muddy and low TDS values. The field absence records of mosquito larvae were distributed across different elevations. This may due to TDS values in water were higher than 1 ppm in moderate-high elevations]. Negative records of mosquito larvae were found in low elevation of coastal regions (<400 m a.s.l.), which received little or no precipitation. This area has high diurnal temperatures (>35°C) during the dry season reducing the probability of available larval mosquito habitats being present due to high evaporation and percolation. The mosquito abundance records collected from field points were statistically associated with different risk levels of the suitable predicted habitats.

Our model indicates that type of land cover and specific environmental attributes are the proxy for predicting *Cx. tritaeniorhynchus* ecological niches. However, our model needs to be validated in other temperate regions where this mosquito vector and viral disease is present.

This model may be useful in delineating the specific ecological variables that are necessary for supporting *Cx. tritaeniorhynchus* larval distribution, but other variables such as urbanization rate, socioeconomic status, association between larval habitats and land cover types, distance to water bodies, land use, and animal shelters may contribute in determining this mosquito ecological niche.

### Conclusion

This study demonstrated that larvae of *Cx. tritaeniorhynchus* preferred aquatic sites with low TDS, wet muddy substrate. These preferable habitats occurred in the areas of hilly middle and eastern Sarwat Mountain range near the cities of Abu Arish, Al Eidaby, Sabya, Ahd Al Masarhah, and Al Ardah. Finally, MaxEnt ecological niche and land cover modeling are useful tools for larval surveillance of *Cx. tritaeniorhynchus*, a vector for RVFV. However, further studies on human demographics, urbanization rate, agricultural development, land use, water use, other interactions with other mosquito vectors, and relative viral transmission need to be addressed in the modeling of RVFV dynamics of the Jazan Province.
