# Correction: Ecological Niche Modeling and Land Cover Risk Areas for Rift Valley Fever Vector, *Culex tritaeniorhynchus* Giles in Jazan, Saudi Arabia

**DOI:** 10.1371/journal.pone.0122908

**Published:** 2015-03-25

**Authors:** The PLOS ONE Staff

There are errors in the Funding section. The correct funding information is as follows: This work is funded by the National Plan for Sciences and Technology through project # 11-ENV-1960-02, King Saud University, Riyadh, KSA. The funders had no role in study design, data collection and analysis, decision to publish, or preparation of the manuscript.
